# Midnolin inhibits coronavirus proliferation by degrading viral proteins

**DOI:** 10.1128/jvi.00366-25

**Published:** 2025-05-29

**Authors:** Yahe Wang, Wu Tong, Wenzhen Qin, Xinyu Yang, Hai Yu, Hao Zheng, Wen Zhang, Guangzhi Tong, Chunmei Wang, Ning Kong, Tongling Shan

**Affiliations:** 1 Shanghai Veterinary Research Institute, Chinese Academy of Agricultural Sciences 12661, Shanghai, China; 2 Jiangsu Co-Innovation Center for the Prevention and Control of Important Animal Infectious Disease and Zoonose, Yangzhou University 38043, Yangzhou, China; 3 School of Medicine, Jiangsu University 191611, Zhenjiang, China; The Ohio State University, Columbus, Ohio, USA

**Keywords:** midnolin, PEDV, proteasome, autophagy, domain

## Abstract

Cells utilize proteasomes and selective autophagy to degrade ubiquitin-labeled viral proteins and inhibit viral proliferation. Midnolin, a midbrain nucleolar protein, is reported to use ubiquitin-independent proteasomal degradation to degrade various nuclear proteins including transcription factors encoded by the immediate-early genes. However, it remains unclear whether midnolin can degrade viral protein to hinder viral replication. In this study, midnolin degraded porcine epidemic diarrhea virus (PEDV) S1/S2/M/E proteins to suppress PEDV proliferation. Midnolin has two essential structural domains: the Catch domain and the ubiquitin-like domain. We found that the Catch domain and the ubiquitin-like domain of midnolin concerted to target and degrade PEDV S1/S2/M/E proteins by both the proteasome and selective autophagy. Furthermore, this study indicated that the individual Catch domain of midnolin degraded PEDV S1/S2/M/E proteins through the autophagy pathway, which recruited the E3 ubiquitinating enzyme MARCH8 to ubiquitinate S1/S2/M/E proteins, and that the ubiquitinated proteins were recognized by Tollip and transported to the lysosome for degradation. Collectively, midnolin degraded viral proteins not only through the proteasomal pathway but also revealed a new mechanism of midnolin-mediated virus restriction, where the Catch domain degraded viral proteins using the midnolin (Catch)-MARCH8-Tollip-autophagosome pathway.

**IMPORTANCE:**

Proteasomes and selective autophagy are two ways that inhibit viral proliferation in cells. Midnolin can degrade nuclear proteins. However, whether midnolin can degrade viral protein is unknown. In this study, we found that midnolin degraded porcine epidemic diarrhea virus (PEDV) S1/S2/M/E proteins to suppress PEDV proliferation. During the degradation, two domains of midnolin exerted a vital role. The Catch domain and the ubiquitin-like domain concerted to interact and degrade PEDV S1/S2/M/E proteins through the proteasome pathway. In addition, the individual Catch domain of midnolin degraded PEDV S1/S2/M/E proteins through the autophagy pathway using the midnolin (Catch)-MARCH8-Tollip-autophagosome pathway. Overall, we have discovered a new mechanism of midnolin which acts as a host factor for antiviral function.

## INTRODUCTION

Coronaviruses (CoVs) are positive-sense single-stranded RNA viruses that infect various hosts, including humans and animals, posing challenges for public health and a veterinary and economic concern (1). CoVs have the largest genome (approximately 27–32 kb) among all known RNA viruses, encoding a methylated cap structure at the 5′ end of the genome, 15 or 16 non-structural proteins, four primary structural proteins (spike protein, S; envelope protein, E; membrane protein, M; and nucleocapsid protein, N), and a poly(A) tail at the 3′ end. Coronaviruses can be divided into four genera (including *Alphacoronavirus*, *Betacoronavirus*, *Gammacoronavirus,* and *Deltacoronavirus*). The former two genera primarily infect mammals, while the latter two predominantly infect birds (2–5). The CoVs genome is extremely susceptible to mutation and can continuously escape the host’s immune response, making it very difficult to prevent and control CoVs.

Porcine epidemic diarrhea virus (PEDV) belonging to the *Alphacoronavirus* genus results in high morbidity and lethality, especially for neonatal piglets, mainly causing severe diarrhea, vomiting, loss of appetite, dehydration, and death (6). At first, PEDV was detected in the UK in 1971 and subsequently spread to a lot of European and Asian countries. In 2010, a PEDV variant strain emerged in Chinese swine farms and spread worldwide, leading to large economic losses (7). The genome of PEDV encodes four structural proteins (S, E, M, and N) (8, 9). The S protein is divided into S1 and S2 subunits. The S1 subunit at the N-terminal of the S protein includes the domain for binding to receptors, while the S2 subunit at the C-terminal is responsible for membrane fusion (10). The S protein plays a vital role in binding and entering the host cell by interacting with its specific receptor, as well as in the formation of syncytia (11). In addition, it stimulates the production of neutralizing antibodies (12). The M protein is a transmembrane protein with high conservation, and it exerts significant functions in viral assembly, germination, and regulation of host immune responses (13, 14). The E protein is also a multifunctional protein participating in the formation of viral capsule membranes and is vital for viral replication and maturation (15, 16).

The balance between protein synthesis and degradation is essential for eukaryotic cell growth and development. Proteasome and autophagy pathways are the primary mechanisms to degrade cellular components (17). Protein degradation makes a critical impact on regulating numerous essential cellular processes including cell cycle, transcription, signaling pathways, intracellular transport, and maintenance of protein quality (18–21). Proteasome degradation is essential in all cells and organisms, and the impairment or breakdown of proteasome degradation has been associated with a range of human diseases (22). Autophagy is a comprehensive process that requires close coordination between different molecular systems. In viral infections, autophagy can enhance or suppress viral replication, depending on the pathogen, host, and cell type (23). For example, the host factor BST2 can degrade PEDV N protein by autophagy (24). During autophagy, the substrate proteins are modified with ubiquitin by E3 ubiquitin ligase MARCH8 and identified through the cargo receptor CALCOCO2. Moreover, these can deliver the substrates and interact with the ATG8 family proteins to form the autophagosome degrading the substrates.

Midnolin is the protein encoded by the MIDN gene. Midnolin was first visualized in the midbrain of mouse embryos with a gene-trapped lacZ insertion (25). In recent years, there has been less research on this gene, and several studies have concentrated on a putative association between MIDN gene copy number reductions and sporadic Parkinson’s disease (26, 27), which has been disputed. Midnolin has been indicated in the ubiquitin-independent recruitment of proteins to the proteasome for degradation, especially targeting transcription factors encoded by immediate-early genes (28). It can directly deliver substrate proteins to the proteasome for degradation without the ubiquitin chains. Midnolin possesses the Catch domain, and this domain consists of two discrete regions, Catch1 and Catch2. The Catch domain is a specific area of the protein responsible for capturing substrate proteins. Ubiquitination-independent degradation is caused by the ubiquitin-like domain of midnolin. However, the antiviral function of midnolin has remained unclear.

Midnolin, as a host factor, degrades coronavirus structural proteins by interacting with PEDV S1/S2/M/E. The degradation occurs through both the proteasome and the selective autophagy pathway. The Catch domain and ubiquitin-like domain of midnolin work to degrade PEDV S1/S2/M/E proteins synergistically. The Catch domain of midnolin exclusively mediates the degradation of PEDV S1/S2/M/E proteins via the autophagy pathway. Both midnolin and S1/S2/M/E interacted with the E3 ubiquitinating enzyme MARCH8 for ubiquitination. Subsequently, the ubiquitinated proteins are recognized by Tollip and transported to the lysosome for degradation. Additionally, this study presented a novel antiviral mechanism of midnolin.

## RESULTS

### Midnolin suppressed PEDV replication in LLC-PK1 cells

**Fig 1:**
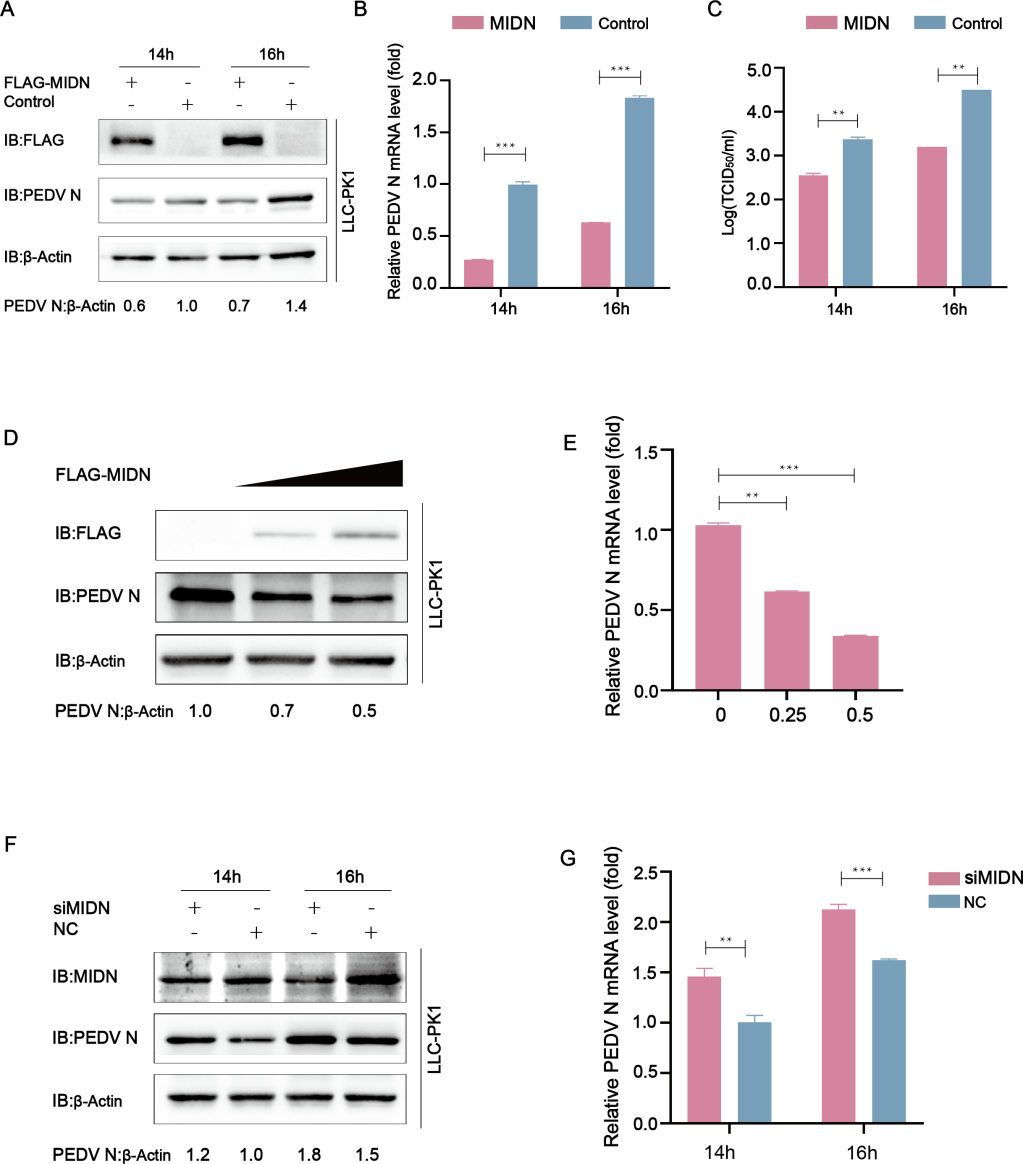
Midnolin hinders PEDV replication in LLC-PK1 cells. (**A–C**) LLC-PK1 cells were subjected to transfection with plasmid encoding Flag-MIDN or the Flag tag and infected with PEDV (MOI = 0.01). Western blot and qRT-PCR were used for analyzing virus replication. Vero cells were subjected to a tissue culture infective dose 50% (TCID_50_) assay. (**D and E**) LLC-PK1 cells were subjected to transfection with Flag-MIDN (wedge) and infected with PEDV (MOI = 0.01). In addition, western blot and qRT-PCR were used to explore virus replication. (**F and G**) LLC-PK1 cells were transfected by MIDN siRNA or negative control siRNA and infected with PEDV (MOI = 0.01). Both western blot and qRT-PCR were used for the analysis of virus replication. Data are shown to be means ± SD of triplicate samples. *P < 0.05, **P < 0.01, ***P < 0.001 (using two-tailed Student’s *t*-test).

To investigate the impact of midnolin on PEDV proliferation, Flag-MIDN and control vector were overexpressed in LLC-PK1 cells. After being transfected for 24 h, cells were infected with PEDV at a multiplicity of infection (MOI) of 0.01. Then, the cells and supernatants were harvested at indicated times. The western blot assay suggested that cells overexpressing MIDN significantly reduced PEDV N protein level compared with those transfected with the control vector (Fig. 1A). Similarly, based on quantitative real-time PCR (qRT-PCR) analysis, the Flag-MIDN group had significantly lower viral mRNA expression (Fig. 1B). Viral titers calculated after infection of Vero cells with supernatant exhibited lower viral titers with MIDN overexpression (Fig. 1C). Moreover, the inhibition effect of midnolin on PEDV replication was dose-dependent (Fig. 1D and E). LLC-PK1 cells were transfected with the siMIDN or negative control siRNA, followed by infection with PEDV at an MOI of 0.01. Cells and supernatants were collected and assayed through western blot and qRT-PCR, respectively. The results demonstrated that interfering with MIDN increased both protein and mRNA levels of PEDV N (Fig. 1F and G). According to the obtained results, midnolin exerted an inhibitory effect on PEDV proliferation.

### Midnolin interacted with PEDV S1/S2/M/E proteins

**Fig 2:**
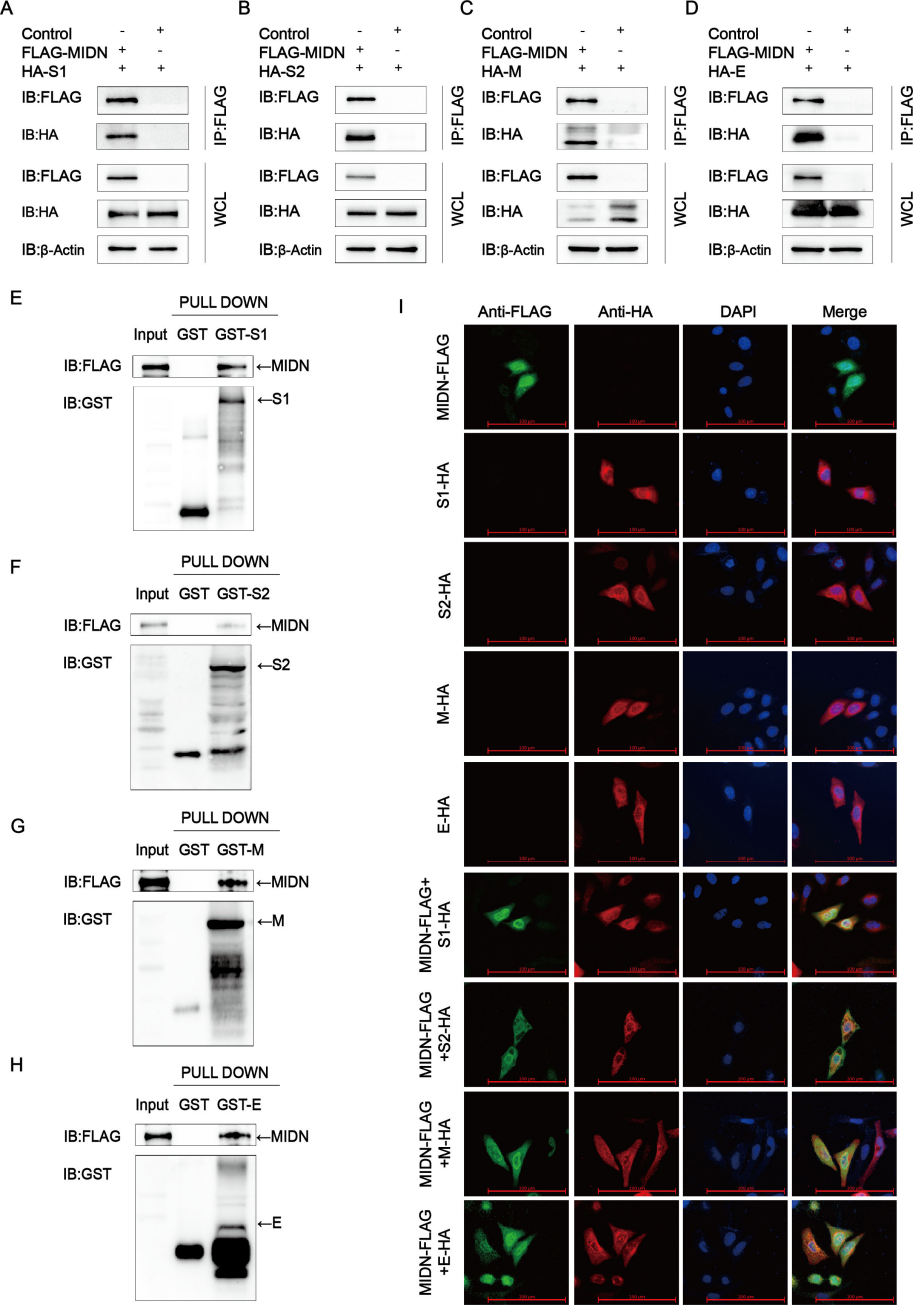
Midnolin makes interactions with PEDV S1/S2/M/E proteins. (**A–D**) Plasmids encoding Flag-MIDN and HA-S1/S2/M/E were transfected into HEK-293T cells for 24 h. Then, the Co-IP assay was used through anti-Flag binding beads, and western blot was carried out for analysis. (**E–H**) MIDN and GST-S1/S2/M/E proteins were expressed by the bacterial strain BL21 (DE3). Using GST pulldown analysis, the correlation between midnolin and the S1/S2/M/E proteins was investigated. (**I**) HeLa cells were transfected with plasmids encoding Flag-MIDN and HA-S1/S2/M/E and labeled with specific primary antibodies and secondary antibodies. In addition, the cell nuclei were stained with 4′,6-diamidino-2-phenylindole (DAPI). The colocalization of midnolin and S1/S2/M/E was found using confocal immunofluorescence microscopy; scale bars: 100 µm.

To further clarify the underlying mechanisms of how midnolin inhibits PEDV proliferation, this study detected whether midnolin interacted with PEDV structural proteins. HEK-293T cells were exposed to co-transfection with HA-S1/S2/M/E and Flag-MIDN plasmids. Coimmunoprecipitation (Co-IP) assay suggested that midnolin efficiently coimmunoprecipitated with PEDV S1/S2/M/E proteins (Fig. 2A through D). A glutathione S-transferase (GST) pulldown assay was conducted to enhance the interaction. The combination of GST-fused S1/S2/M/E (GST-S1/S2/M/E) with midnolin was observed, while no such combination was found between GST and midnolin (Fig. 2E through H). The colocalization of HA-S1/S2/M/E and Flag-MIDN was observed in HeLa cells using confocal microscopy (Fig. 2I). Based on these experiments, midnolin could interact with PEDV S1/S2/M/E proteins.

### Midnolin promoted PEDV S1/S2/M/E protein degradation by autophagy and the proteasome pathway

**Fig 3:**
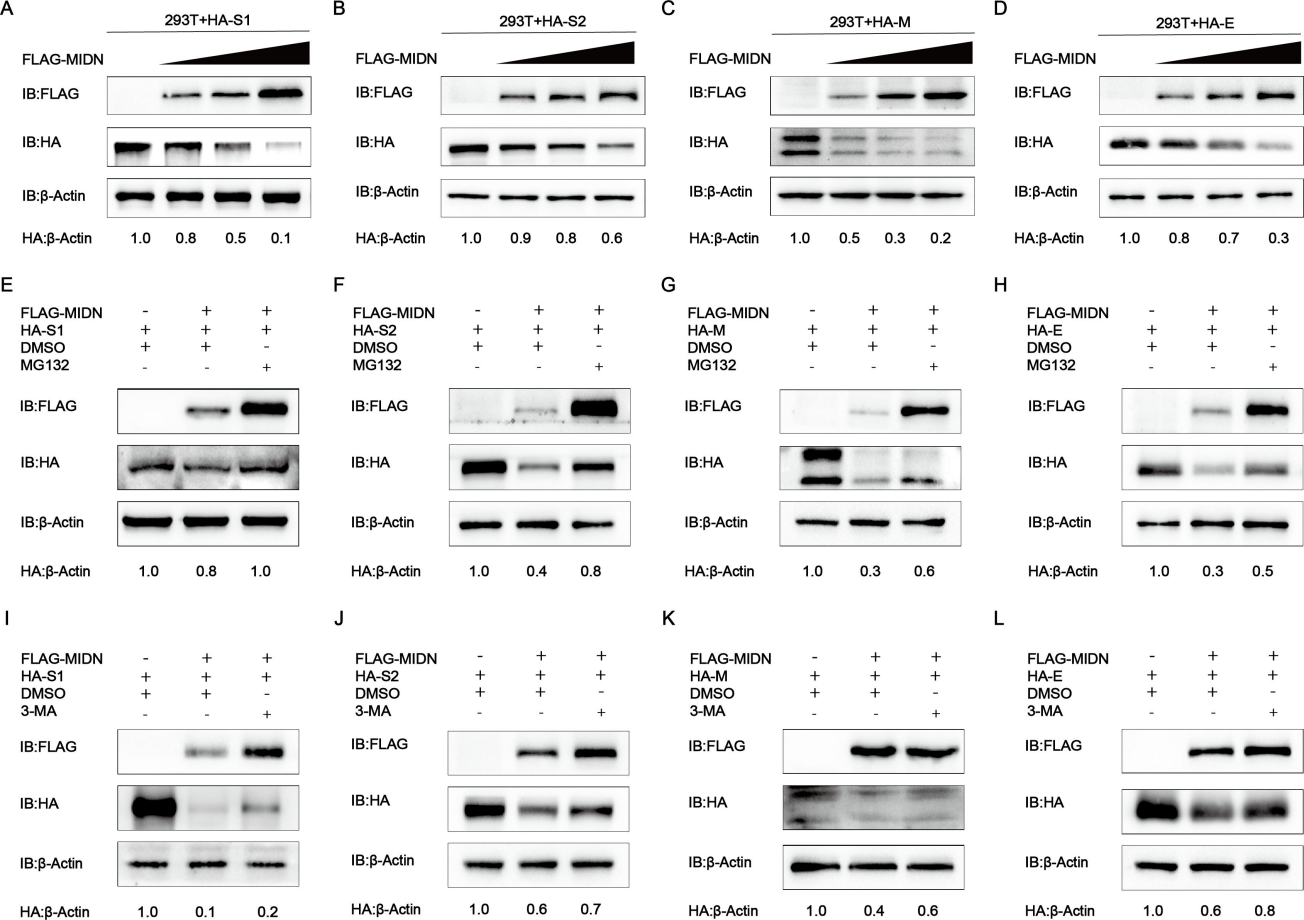
Midnolin degrades PEDV S1/S2/M/E by proteasome and autophagy. (**A–D**) The HA-S1/S2/M/E expression vector and Flag-MIDN expression vector (wedge) were transfected into HEK-293T cells. Western blot was used for the analysis of the cell lysates. β-Actin served as a sample loading control. (**E–L**) The Flag-MIDN and HA-S1/S2/M/E expression vectors were transfected into HEK-293T cells, and cells were subsequently exposed to treatment with MG132 (5 mM) or 3-MA (1 mM) for 9 h. Western blot was used for the analysis of the cell lysates.

Since midnolin is correlated with the proteasome to stimulate the degradation of many nuclear proteins through a ubiquitination-independent mechanism (28), we speculated that its capability of hindering PEDV replication was also caused by the degradation of viral structural proteins. To confirm this hypothesis, HEK-293T cells were co-transfected with HA-S1/S2/M/E and different doses of Flag-MIDN plasmids. To investigate whether midnolin can degrade PEDV structural proteins, western blot analysis indicated that the expression of midnolin decreased the abundance of PEDV S1/S2/M/E proteins, and the inhibition was dose-dependent (Fig. 3A through D). Protein degradation in eukaryotic cells occurs through two main mechanisms, including proteasome and autophagy (29). To examine whether midnolin degrades PEDV structural proteins by proteasome and autophagy pathways, HA-S1/S2/M/E and Flag-MIDN plasmids were co-transfected into HEK-293T cells. After 24 h, MG132, an inhibitor of the proteasome pathway, was added to the cells. The western blot result showed that the degradation of S1/S2/M/E protein mediated by midnolin was blocked by the proteasome inhibitor MG132 (Fig. 3E through H), which is consistent with the previous description that midnolin can degrade proteins through the proteasome pathway. In addition to the proteasome pathway, we also investigated whether midnolin could degrade PEDV structural proteins through autophagy. Different from the above experiments, the autophagy inhibitor 3-methyladenine (3-MA) was added in the cell culture supernatant. Interestingly, we found that the degradation of PEDV S1/S2/M/E proteins mediated by midnolin was also blocked by the autophagy inhibitor 3-MA (Fig. 3I through L), suggesting that midnolin also degraded PEDV S1/S2/M/E proteins through the autophagy pathway. Our results suggested that midnolin degraded PEDV S1/S2/M/E proteins not only through the proteasome pathway but also via the autophagy pathway.

### The Catch domain exerted a vital role in the degradation of midnolin

**Fig 4:**
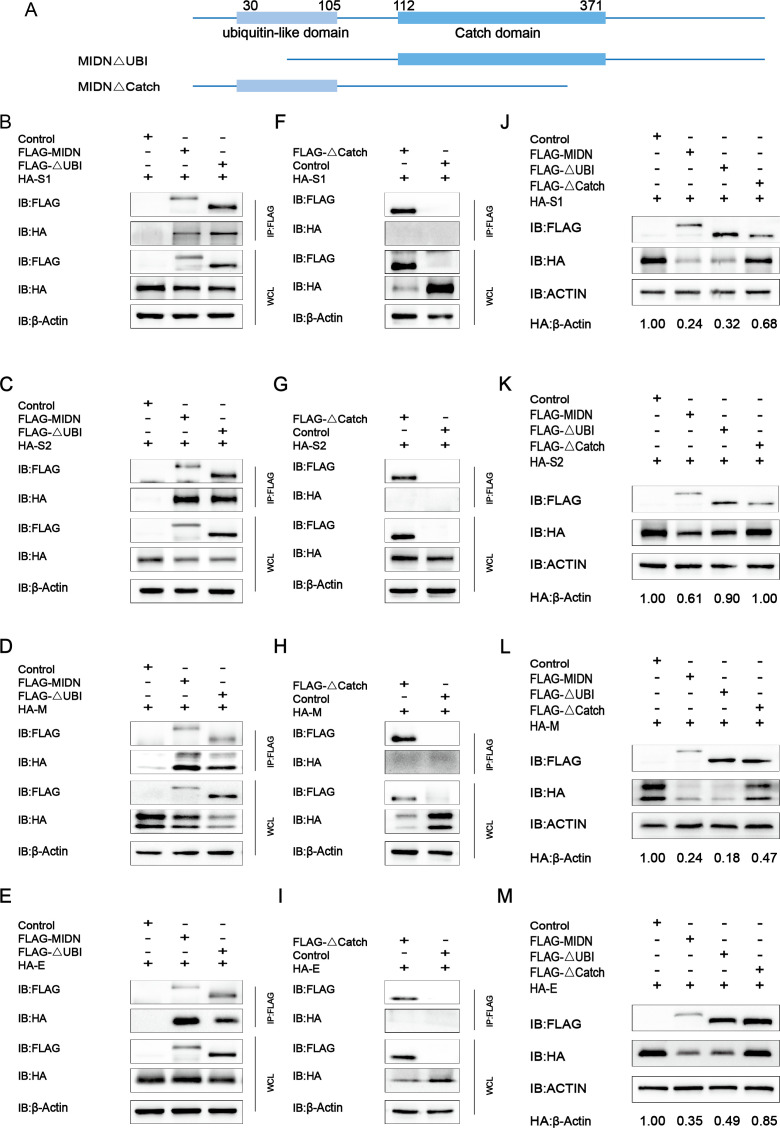
Interaction and degradation of midnolin truncation mutants with PEDV S1/S2/M/E proteins. (**A**) Segmented truncation pattern diagram of midnolin. (**B–E**) HEK-293T cells were transfected with plasmids encoding HA-S1/S2/M/E and Flag-MIDN or Flag-MIDN△UBl. Then, they were explored with Co-IP with anti-Flag binding beads. The whole process was the western blot analysis of whole-cell lysates (WCLs) without immunoprecipitation. (**F–I**) HEK-293T cells were transfected with plasmids encoding HA-S1/S2/M/E and Flag-MIDN△Catch. Then, they were explored with Co-IP with anti-Flag binding beads. The whole process was the western blot analysis of WCLs without immunoprecipitation. (**J–M**) HEK-293T cells were exposed to transfection with the vector which expressed HA-S1/S2/M/E and Flag-MIDN or the indicated MIDN mutants for a day. The cell lysates were explored using western blot. β-Actin acted as the sample loading control.

Midnolin contains different domains including the Catch domain and ubiquitin-like domain. Each domain exerts different functions. For instance, two Catch domains forming a symmetrical hydrophobic zone play an essential role in capturing the substrate proteins for degradation. The ubiquitin-like domain allows the substrate proteins to be degraded by the proteasome without a ubiquitin chain. To explore whether the degradation effect of midnolin on PEDV structural proteins is related to the different domains, we constructed two mutants with deletion of the different domains, respectively: MIDN△Ubl, MIDN△Catch (Fig. 4A). Then, coimmunoprecipitation experiments were performed to identify the correlation between PEDV S1/S2/M/E proteins and midnolin structural domain deletion mutants. HA-S1/S2/M/E and Flag-MIDN△Ubl or Flag-MIDN△Catch were co-transfected in HEK-293T cells, respectively. It was discovered that the deletion of the Ubl domain was associated with PEDV S1/S2/M/E proteins, while that of the Catch domain was unable to interact with those proteins (Fig. 4B through I), suggesting that the Catch domain was essential for the interaction between midnolin and PEDV viral proteins. The degradation of S1/S2/M/E protein mediated by midnolin was blocked by the proteasome inhibitor MG132 (Fig. 3J through M), indicating that midnolin degraded viral proteins via the proteasome pathway. We overexpressed Flag-Ubl in LLC-PK1 cells, implying that the Ubl domain was unable to affect PEDV replication. And we overexpressed Flag-Ubl and HA-S1/S2/M/E plasmids in HEK-293T cells, implying that the Ubl domain was unable to coimmunoprecipitate with PEDV S1/S2/M/E proteins (data not shown). Therefore, we speculated that Ubl and Catch domains worked together to target PEDV S1/S2/M/E proteins via proteasome for degradation, and that the protein degradation pathways are consistent as previously described. Next, we investigated the impact of midnolin deletion domain mutants on PEDV S1/S2/M/E proteins degradation, finding that the degradation of S1/S2/M/E proteins by midnolin was significantly reduced after the deletion of the Catch domain (Fig. 4F through I). Therefore, it is suggested that the Catch domain of midnolin occupies an essential position in the function of PEDV S1/S2/M/E proteins degradation.

### The Catch domain of midnolin degraded PEDV S1/S2/M/E through autophagy

**Fig 5:**
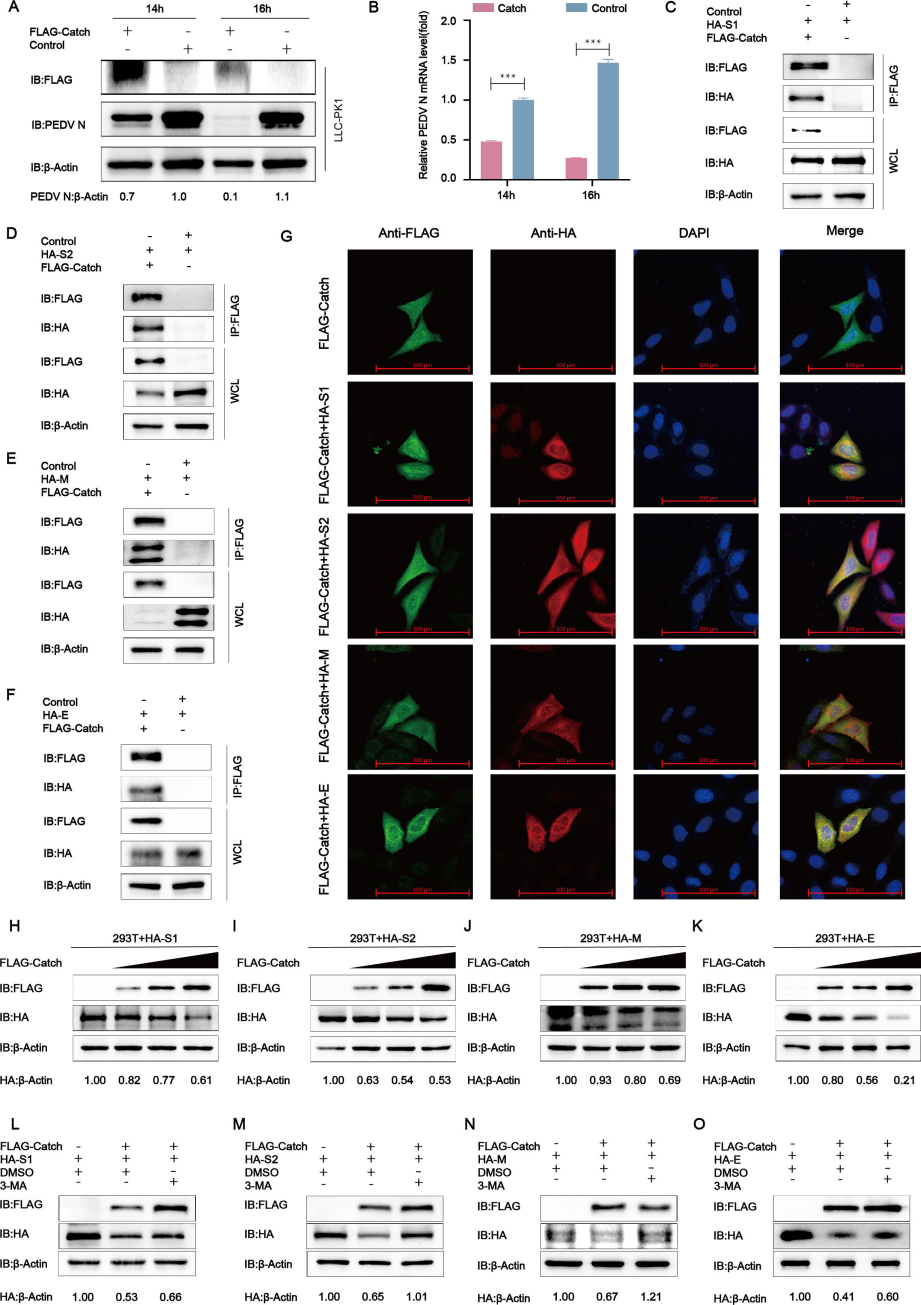
The Catch domain of midnolin interacts with PEDV S1/S2/M/E and degrades PEDV S1/S2/M/E by autophagy. (**A and B**) LLC-PK1 cells were transfected with a plasmid encoding Flag-Catch or the Flag tag, infected with PEDV (MOI = 0.01), and then harvested at indicated times. Western blot and qRT-PCR were employed to explore cell lysates; β-actin acted as the sample loading control. (**C–F**) Flag-Catch and HA-S1/S2/M/E were transfected into HEK-293T cells. Co-IP assay was adopted for exploring the interaction between the Catch domain and HA-S1/S2/M/E protein. (**G**) HeLa cells were exposed to transfection with Flag-Catch and HA-S1/S2/M/E, and subsequently labeled with specific primary antibodies and secondary antibodies. The cell nuclei were stained with 4′,6-diamidino-2-phenylindole (DAPI). Then, the colocalization of Catch and S1/S2/M/E was observed with confocal immunofluorescence microscopy; scale bars: 100 µm. (**H–K**) The HA-S1/S2/M/E expression vector and Flag-Catch expression vector (wedge) were exposed to transfection into HEK-293T cells. Western blot was adopted for the analysis of the cell lysates. β-Actin served as a sample loading control. (**L–O**) The Flag-Catch and HA-S1/S2/M/E expression vectors were transfected into the HEK-293T cells for 24 h, followed by the treatment with 3-MA (1 mM) for 9 h. Afterward, the cell lysates were investigated using western blot.

As the midnolin degraded PEDV S1/S2/M/E proteins through the proteasome pathway and autophagy pathway (Fig. 3), and the Catch domain of midnolin makes a vital impact on the function of PEDV S1/S2/M/E protein degradation (Fig. 4), we speculated that the Catch domain degraded viral proteins through the autophagy pathway. In addition, we overexpressed Flag-Catch in LLC-PK1 cells, concluding that the replication of PEDV was significantly inhibited in the Catch domain overexpressing cells (Fig. 5A and B). Next, it was found that the Catch domain interacted with PEDV S1/S2/M/E proteins (Fig. 5C through F). Consistent with the obtained results, the colocalization of S1/S2/M/E and Catch was observed with confocal immunofluorescence assay (Fig. 5G). To investigate whether the Catch domain can degrade PEDV structural proteins, HEK-293T cells were co-transfected with HA-S1/S2/M/E and different doses of Flag-Catch plasmids. Based on western blot analysis, the expression of the Catch domain reduced the abundance of PEDV S1/S2/M/E proteins, and the inhibition was dose-dependent (Fig. 5H through K). To further verify the degradation through the autophagy pathway, the autophagy inhibitor 3-MA was added in HA-S1/S2/M/E and Flag-Catch plasmids co-transfected cells. Western blot assay showed that the degradation of PEDV S1/S2/M/E by the Catch domains was significantly inhibited by 3-MA (Fig. 5L through O). These results suggested that the Catch domain of midnolin degraded PEDV S1/S2/M/E through the autophagy pathway.

### The midnolin (Catch)-MARCH8-Tollip-autophagosome pathway degraded PEDV S1/S2/M/E proteins

**Fig 6:**
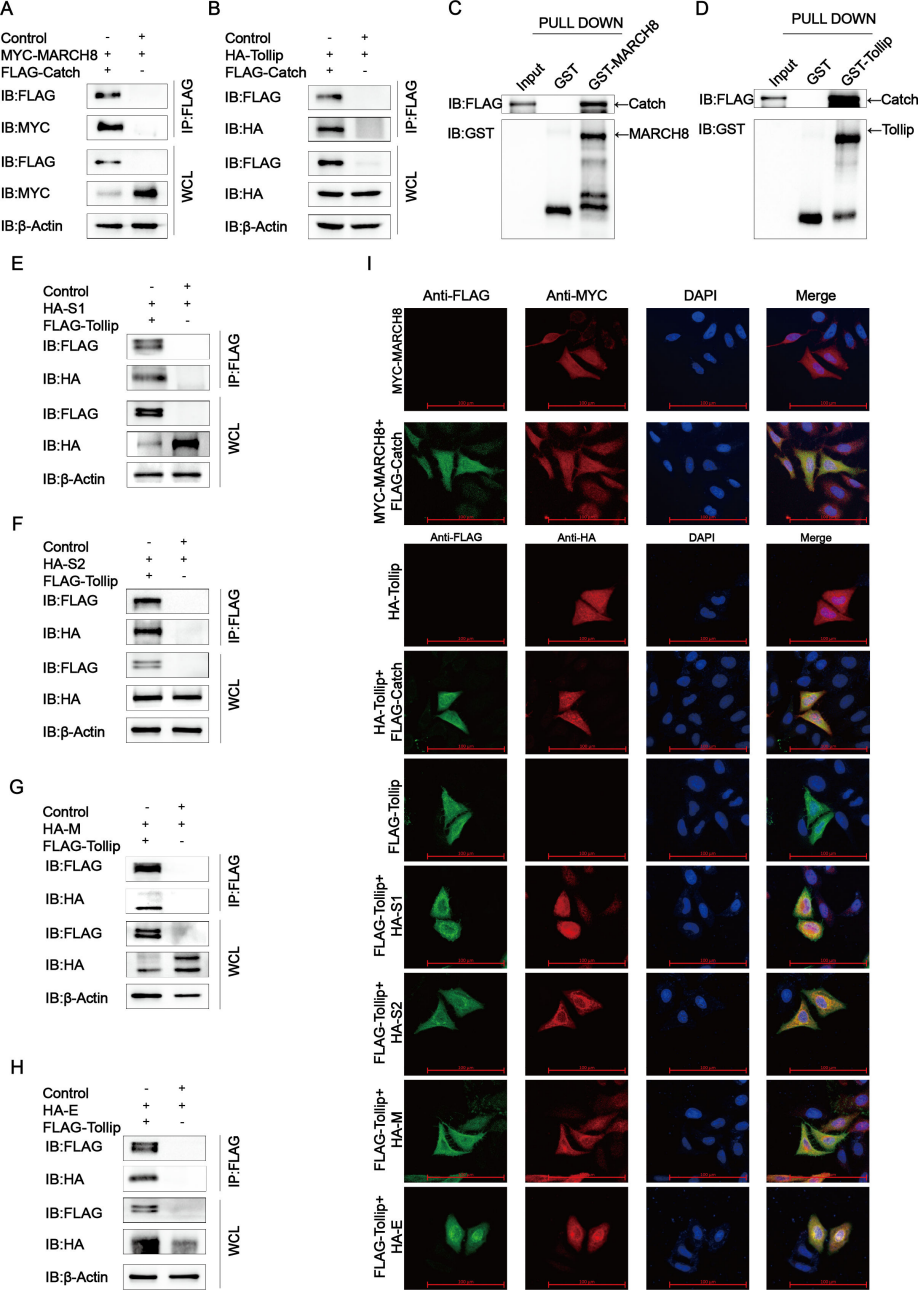
PEDV S1/S2/M/E proteins are degraded by autophagy via interaction with MARCH8 and Tollip. (**A and B**) HEK-293T cells were subjected to transfection with plasmids encoding Flag-Catch and MYC-MARCH8 or HA-Tollip for 24 h and subsequently explored with Co-IP assay. β-Actin was used as the sample loading control. (**C and D**) The MARCH8 and Tollip were cloned into pCold TF plasmid and Catch was cloned into pCold GST plasmid, respectively. Recombinant proteins were indicated in bacterial strain BL21 (DE3) and purified for the GST pulldown analysis. (**E–H**) HEK-293T cells were transfected with plasmids encoding Flag-Tollip and HA-S1/S2/M/E for 24 h and subsequently explored with Co-IP assay. β-Actin served as the sample loading control. (**I**) HeLa cells were exposed to transfection with plasmids encoding Flag-Tollip and HA-S1/S2/M/E, Flag-Catch and MYC-MARCH8, or Flag-Catch and HA-Tollip. Additionally, the cell nuclei were stained with 4′,6-diamidino-2-phenylindole (DAPI). Meanwhile, the colocalization of Tollip and S1/S2/M/E, Catch and MARCH8, or Catch and Tollip was observed with the use of confocal immunofluorescence microscopy; scale bars: 100 µm.

**Fig 7:**
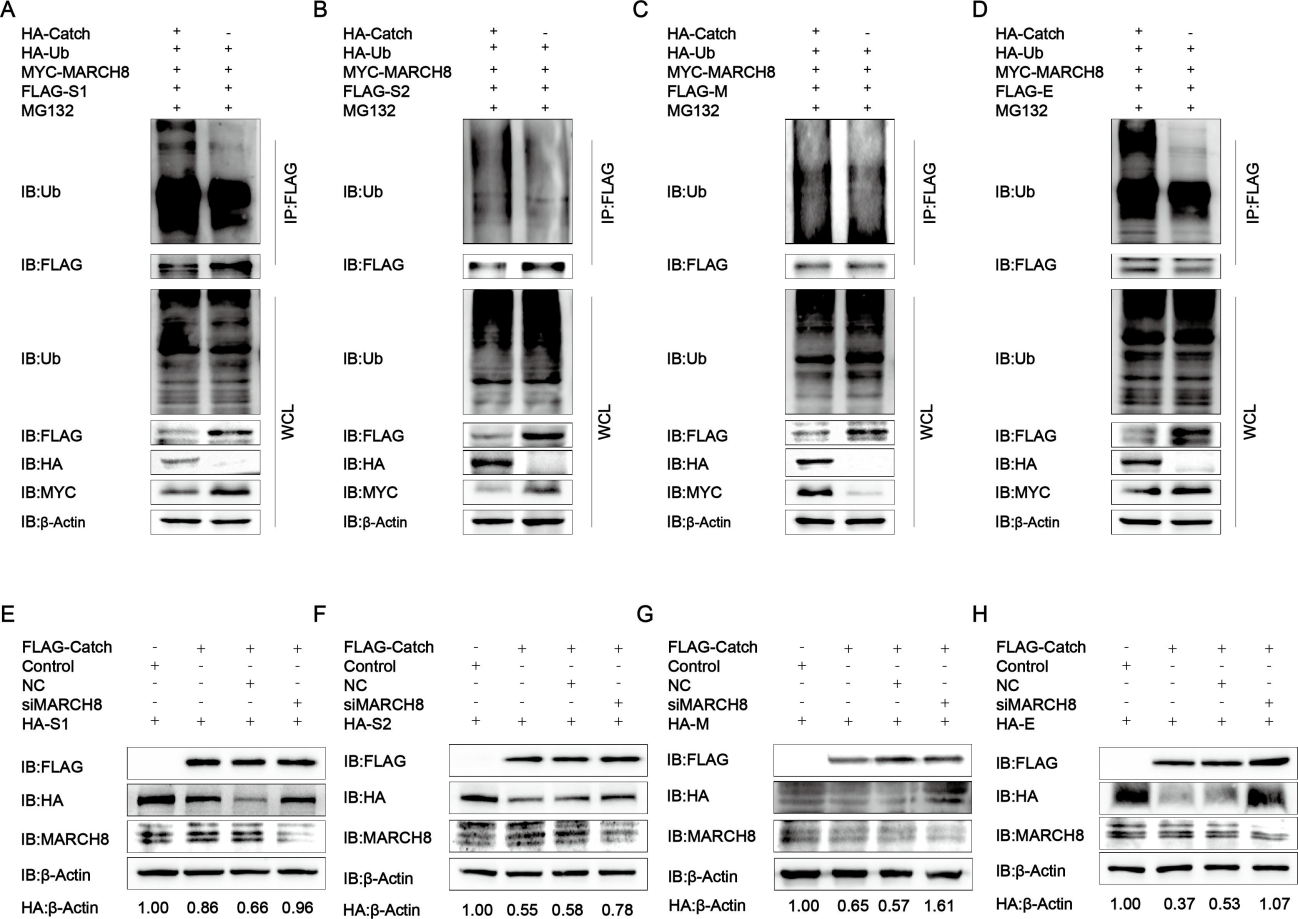
PEDV S1/S2/M/E proteins are degraded by autophagy through the midnolin (Catch)-MARCH8-Tollip-autophagosome pathway. (**A through D**) HEK-293T cells co-transfected with Flag-S1/S2/M/E and HA-Catch plasmids and cellular lysates collected after subjecting to post-transfection for 24 h. The ubiquitinated S1/S2/M/E proteins were exposed to immunoprecipitation with an anti-Flag antibody. All the involved samples were explored with the application of western blotting. (**E through H**) HEK-293T cells were exposed to co-transfection using plasmids encoding Flag-Catch and HA-S1/S2/M/E and small-interfering RNA (MARCH8 siRNA or negative control siRNA) and subsequently explored with western blot.

Ubiquitination of the substrate protein by E3 ubiquitin ligase is the first step in autophagy, followed by the recognition and delivery by the cargo receptor to the lysosome for degradation. It was previously found that the host factor BST2 recruited MARCH8 to catalyze the ubiquitination of the PEDV N protein and degraded the ubiquitinated N protein through the selective autophagy pathway (24). To test whether MARCH8 is engaged in the adjustment of PEDV S1/S2/M/E protein stability mediated by the Catch domain of midnolin, this study examined the MARCH8-Catch domain association, finding that MARCH8 made interactions with the Catch domain in HEK-293T cells (Fig. 6A). This indicated an underlying regulatory role of MARCH8 in the Catch domain-mediated PEDV structural protein degradation. A growing body of evidence demonstrated that cargo receptors made vital impacts on delivering substrates to the autophagosome for selective degradation (29). We transfected the commonly used cargo receptors including SQSTM1/p62 (sequestosome 1), OPTN (optineurin), CALCOCO2/NDP52 (calcium binding and coiled-coil domain 2), and Tollip (toll interacting protein) (30) into HEK-293T cells, concluding that the Catch domain of midnolin was related to Tollip rather than other cargo receptors (Fig. 6B). Consistent with the obtained results, the interaction of the Catch domain of midnolin and MARCH8 and Tollip was also confirmed by the GST pulldown assay and confocal immunofluorescence assay (Fig. 6C, D and I). To further demonstrate that Tollip was involved in Catch domain-induced PEDV structural protein degradation, Flag-Tollip and HA-S1/S2/M/E plasmids were co-transfected into HEK-293T cells. Co-IP and confocal immunofluorescence assays indicated that Tollip interacted with PEDV S1/S2/M/E proteins (Fig. 6E through I). These data suggested that the Catch domain of midnolin linked the PEDV S1/S2/M/E proteins and recruited MARCH8 to the cargo receptor Tollip for autophagic degradation. We overexpressed Flag-Catch in the MYC-MARCH8 and HA-S1/S2/M/E-overexpressing HEK-293T cells to verify whether MARCH8 can ubiquitinate S1/S2/M/E proteins. Based on the results of the western blot, the simultaneous presence of MARCH8 and Catch can significantly enhance the ubiquitination of S1/S2/M/E (Fig. 7A through D). To further substantiate that the MARCH8-Tollip-autophagosome pathway is essential for the Catch domain-induced degradation of S1/S2/M/E proteins, we interfered with the degradation pathway by siMARCH8, finding that the Catch domain-induced PEDV S1/S2/M/E degradation was abolished in MARCH8-interfering cells (Fig. 7E through H). All of these data suggested that the Catch domain of midnolin degraded PEDV S1/S2/M/E proteins by autophagy via the midnolin (Catch)-MARCH8-Tollip-autophagosome axis.

### Midnolin suppressed CoV replication in LLC-PK1 cells

**Fig 8:**
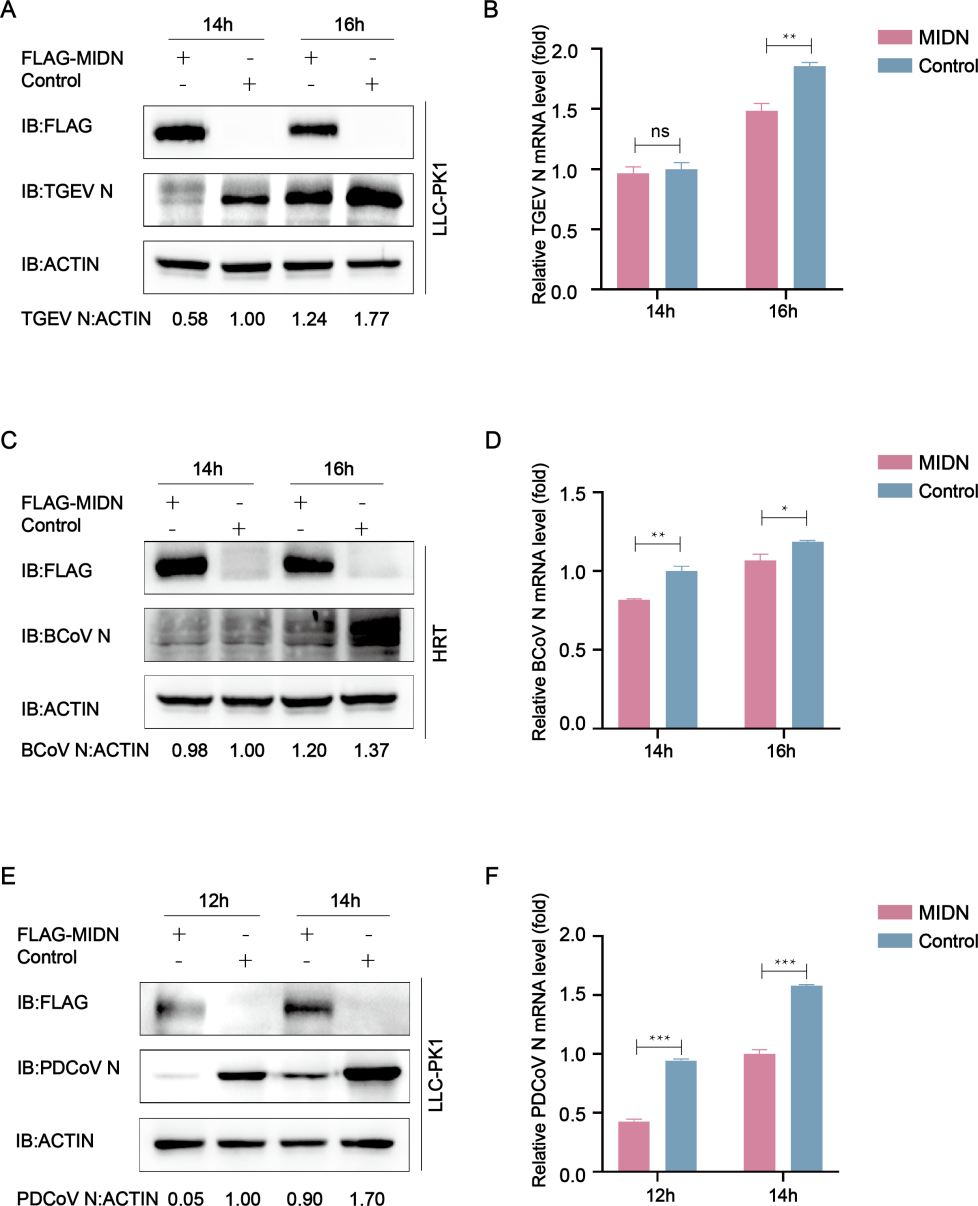
Midnolin inhibits coronavirus replication in LLC-PK1 cells. (**A and B**) LLC-PK1 cells were subjected to transfection with a plasmid encoding Flag-MIDN or the Flag tag. Afterward, the cells were infected with TGEV and then harvested at indicated times. Western blot and qRT-PCR were used for analyzing cell lysates. β-Actin acted as the sample loading control. (**C and D**) LLC-PK1 cells were transfected with a plasmid encoding Flag-MIDN or the Flag tag. Next, the cells were infected with BCoV and harvested at indicated times. Western blot and qRT-PCR were employed to investigate cell lysates. (**E and F**) LLC-PK1 cells were transfected with a plasmid encoding Flag-MIDN or the Flag tag. Subsequently, the cells were infected with PDCoV and harvested at indicated times. The analysis of the cells was performed by the analysis of western blot and qRT-PCR.

The CoVs can be classified into four groups including *alpha, beta, gamma*, and *delta* by phylogenetic clustering (3). Our previous study revealed that midnolin inhibited PEDV replication, and thus we speculated that midnolin might exert a general inhibitory effect on coronavirus. LLC-PK1 cells or HRT cells were transfected with Flag-MIDN plasmid and infected with transmissible gastroenteritis virus (TGEV) (*Alphacoronavirus*), Bovine coronavirus (BCoV) (*Betacoronavirus*), or porcine delta coronavirus (PDCoV) (*Deltacoronavirus*). Using the western blot assay, it was indicated that cells overexpressing midnolin significantly reduced N protein levels of these different viruses compared with those transfected with the control vector (Fig. 8A, C and E). Consistent with this observation, qRT-PCR analysis suggested that the Flag-MIDN group had significantly lower viral mRNA expression (Fig. 8B, D and F). Moreover, these results demonstrated that midnolin had the capacity to suppress coronavirus.

## DISCUSSION

**Fig 9:**
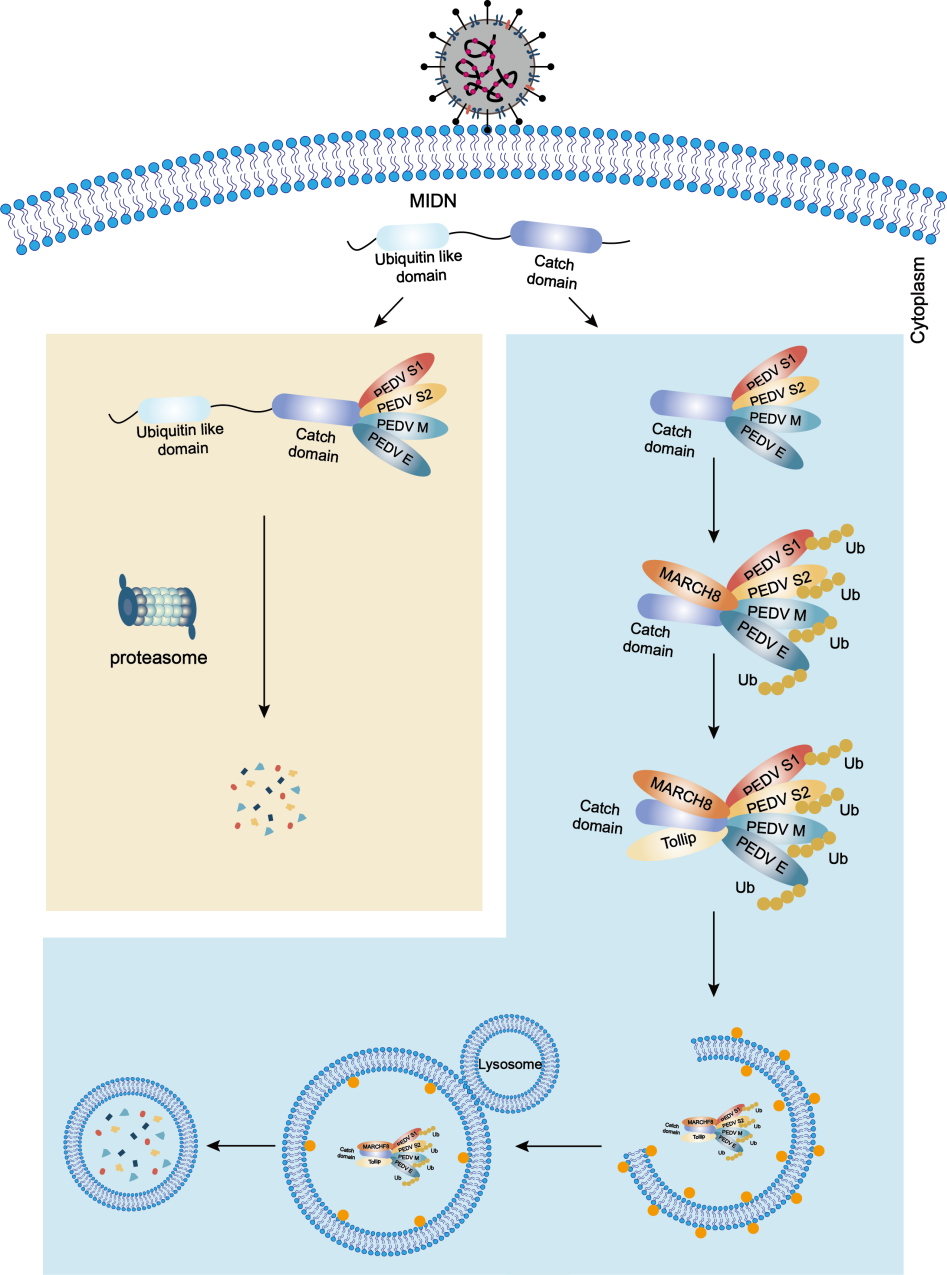
The antiviral mechanism of midnolin inhibits PEDV replication. Midnolin has two vital structural domains, including the Catch domain and the ubiquitin-like domain. During the period of PEDV infection, the Catch domain and ubiquitin-like domain of midnolin concerted to degrade PEDV S1/S2/M/E proteins through the proteasome pathway. At the same time, the Catch domain recruited E3 ubiquitin ligase MARCH8 to catalyze PEDV S1/S2/M/E proteins. The ubiquitinated viral proteins were detected and degraded through Tollip-dependent selective autophagy.

Midnolin is a newly discovered protein whose protein function is unclear. Recently, midnolin has been shown to be indicated in the ubiquitin-independent recruitment of proteins to the proteasome for degradation, especially targeting transcription factors encoded by immediate-early genes (28). This study has identified a novel mechanism through which midnolin modulates the proliferation of coronavirus. Midnolin interacted with PEDV S1/S2/M/E proteins and degraded these proteins through both proteasome and autophagy pathways to hinder the replication of PEDV. The midnolin Ubl domain and Catch domain are vital for its degradation function. The Ubl domain and the Catch domain together targeted and degraded viral proteins through the proteasome, while only the Catch domain degrades viral proteins via the Catch-MARCH8-Tollip-autophagosome axis (Fig. 9).

CoVs are enveloped viruses with a single-stranded, positive-sense RNA genome. The CoV that infects humans can cause acute respiratory symptoms, including severe acute respiratory syndrome coronavirus (SARS-CoV), Middle East respiratory syndrome coronavirus (MERS-CoV), and SARS-CoV-2 (31). CoV infecting swine can cause acute gastrointestinal symptoms, including PEDV, TGEV, PDCoV, and swine acute diarrhea syndrome coronavirus (32, 33). CoVs can cause significant harm to the pig industry (34). Therefore, it is urgent to prevent and control virus transmission. In this study, it was observed that midnolin effectively suppressed the replication of various coronaviruses, encompassing α-CoV PEDV and TGEV, β-CoV BCov, and δ-CoV PDCov. This implies that midnolin exerts a wide-ranging inhibitory impact on coronaviruses. The majority of coronaviruses have coronal processes that are formed by the trimeric S protein (35). The S protein can primarily mediate viral invasion and determine viral tissue or host tropism by binding to host cell receptors. The envelope is mainly composed of the M protein (2). The M protein is part of the viral envelope and can be engaged in the assembly and release of viral particles (36). E protein is present at low levels in the envelope. The E protein influences the immune response of the host, therefore regulating PEDV infection. An interaction between midnolin and the PEDV S1/S2/M/E proteins was observed in this study. Moreover, midnolin has been shown to induce significant degradation of the PEDV S1/S2/M/E proteins. As a result, it can be speculated that the new function of the midnolin-mediated viral restriction mechanisms probably depends on the degradation of viral nucleocapsid protein.

The interaction of viral and host factors determines the vulnerability to viral infection and the development of pathogenesis (37). The precise mechanisms through which these factors exert their influence on pathogenesis play a vital role in formulating strategies aimed at the prevention of infection (38). Our previous study showed that host factors including TARDBP, FUBP3, HNRNPA1, PTBP1, PGAM5, and RBM14 degraded coronavirus structural proteins through selective autophagy or proteasome to inhibit the proliferation of coronaviruses (39–44). The autolysosome and proteasome pathways are the primary protein degradation mechanisms in eukaryotic cells. In this study, we found that the addition of proteasome inhibitors and autophagy inhibitors effectively suppressed the degradation of PEDV structural proteins by midnolin. This implies that midnolin facilitates the degradation of viral proteins via both the proteasome and autophagy pathways.

Initially, midnolin was detected due to the strong induction in the midbrain during early embryonic development, and it is believed that the biological function of midnolin may be multifaceted. Considering that many of its substrates make vital impacts on both the nervous and immune systems, clarifying the physiological role of midnolin in the body holds significant importance. Midnolin contains the Catch domain and ubiquitin-like domain, which exert different roles in the degradation of proteins. In this study, the absence of the Catch domain of midnolin resulted in a loss of interaction with PEDV S1/S2/M/E proteins, and the degradation capacity of midnolin was significantly compromised. After amplifying the ubiquitin-like domain and Catch domain, respectively, it was observed that the ubiquitin-like domain alone was unable to inhibit PEDV proliferation or interact with PEDV structural proteins. They demonstrated that the Catch domain played a significant role in the degradation.

Autophagy is a fundamental eukaryotic pathway exerting different impacts on immunity (45). A growing body of evidence points to the crosstalk between autophagy and virus replication (46). Autophagy exerts a dual role in the context of viral infection. Host cells induce autophagy as a defense mechanism to eliminate viral particles. Additionally, viruses exploit autophagosomes to create a membrane-bound niche supporting their self-replication by providing essential metabolites and energy (47, 48). This study found that only the Catch domain of midnolin was inhibited in PEDV replication, and the ability to inhibit virus replication was inhibited by the autophagy inhibitor, suggesting that the Catch domain of midnolin could inhibit PEDV replication by autophagy. The process of autophagy involves the selective targeting and degradation of specific substrates. In selective autophagy, these specific substrates can be ubiquitinated by E3 ubiquitin ligase and recognized by cargo receptors (49). We indicated that the Catch domain recruited E3 ubiquitin ligase MARCH8 for the catalysis of PEDV S1/S2/M/E proteins, and the ubiquitinated S1/S2/M/E proteins were recognized and degraded by Tollip-dependent selective autophagy.

Collectively, this study determined the additional role of midnolin as a negative regulator in CoV replication. The obtained results suggested that a working model was proposed to clarify how midnolin exerts the antiviral role in virus replication: midnolin can degrade PEDV S1/S2/M/E proteins to suppress virus proliferation by both proteasome and selective autophagy pathways. The Catch domain and Ubl domain of midnolin made concerted efforts to degrade PEDV S1/S2/M/E proteins through the proteasome pathway. Meanwhile, only the Catch domain was capable of recruiting E3 ubiquitin ligase MARCH8 to catalyze PEDV S1/S2/M/E proteins, and the ubiquitinated viral proteins were identified and degraded by Tollip-dependent selective autophagy. The finding provides evidence to support the antiviral functions of midnolin in CoV replication via targeting and degrading the viral proteins through the proteasome and selective autophagy.

## MATERIALS AND METHODS

### Antibodies and reagents

Anti-MIDN antibody (18939-1-AP) and anti-HA (HRP-81290) were purchased from Proteintech. Anti-ubiquitin antibody (SC-8017) was obtained from Santa Cruz Biotechnology. Anti-ACTB antibody (60,008-1), anti-GST-tag antibody (10,000-0-AP), and anti-MARCH8 antibody (14,119-1-AP) were purchased from the Proteintech Group. In addition, anti-FLAG antibody (SB-AB0008), horseradish peroxidase (HRP)-conjugated anti-mouse IgG antibody (SB-AB0102), and HRP-conjugated anti-rabbit IgG (SB-AB0101) antibody were purchased from ShareBio. The preparation of monoclonal antibody against PEDV N protein was performed in our laboratory (50). 3-MA (M9281) and MG132 (M7449) were purchased from Sigma-Aldrich. Following the instructions of the manufacturer, all the recombinant plasmids were built by homologous recombination with the ClonExpress II One Step Cloning Kit (Vazyme Biotech, C112- 02).

### Cell cultures and transfection

Human embryonic kidney cells (HEK-293T cells; ATCC, CRL11,268) were cultivated within Dulbecco's modified Eagle medium (DMEM) (Sigma-Aldrich, D6429) supplemented with the concentration of 10% FBS. Porcine kidney cells (LLC-PK1 cells) were obtained from Dr. Rui Luo (Huazhong Agricultural University, Wuhan, China) and maintained in modified Eagle’s medium (Invitrogen, 11,095,080). All cells were subject to incubation at 37°C and 5% CO_2_. Subsequently, cells grown to approximately 80%–90% confluence were transfected with plasmids with Lipofectamine 3000 Reagent (Invitrogen, L3000015). After the relevant instructions, the cells grown to 50%–60% confluence were subjected to transfection with siRNA based on Lipofectamine RNAiMAX (Invitrogen, 13778150). The PEDV variant strain JS-2013, TGEV, BCoV, and PDCoV were isolated and preserved in our laboratory.

### Quantitative real-time PCR (qPCR)

The extraction of total RNA was performed from cells with the Vazyme Viral RNA Mini Kit (RC311-01). For the reverse transcription (RT)-qPCR analysis, cDNA was generated using the HiScript RT SuperMix for qPCR (Vazyme, R123-01) and explored using qPCR with SYBR qPCR Master Mix (Vazyme, Q712-02) in line with the instructions of the manufacturer. GAPDH served as the reference gene for normalization.

### Western blotting analysis

Cells were subjected on ice with RIPA Lysis and Extraction Buffer (Thermo Fisher Scientific, 89901) including Protease Inhibitor Cocktail (Bimake, B14001). Then, the lysates were denatured for 10 min within 5 × SDS-PAGE loading buffer and later separated with SDS-PAGE. Next, the proteins were transferred to nitrocellulose western blotting membranes (GE Healthcare, 10600001). The membranes were blocked with phosphate buffered saline (PBS) containing 5% nonfat dry milk (NB1172-100G) and 0.2% Tween 20 (Sigma-Aldrich, P1379) for 60 min at room temperature, and subsequently incubated with the primary antibody at room temperature for 60 min. When the membranes were rinsed with PBS including 0.1% Tween 20 (0.1% PBST), they were exposed to incubation with the corresponding secondary antibody for 60 min and identified using enhanced chemiluminescence (Beyotime, P0209). The ImageJ software (National Institutes of Health) was used for the quantification of the protein bands.

### Co-IP assay

Cells were exposed to transfection with the indicated plasmids for 24 h and then lysed with ice-cold NP40 Cell Lysis Buffer (Life Technologies, FNN0021) including Protease Inhibitor Cocktail. Next, the cell lysates were exposed to centrifugation and subjected to incubation with anti-Flag-antibody-bound Dynabeads Protein G (Life Technologies, 10004D) for 30 min at room temperature. Next, the Dynabeads were rinsed four times with 0.02% PBST and resuspended in elution buffer (50 mM glycine, pH 2.8). Based on the indicated antibodies, proteins were explored with the application of immunoblotting.

### GST affinity isolation assays

The full-length *MIDN*, PEDV *S1/S2/M/E*, *MARCH8,* and *Tollip* were cloned into pCold GST plasmid (Clontech Laboratories, Inc., 3372) or pCold TF plasmid (Clontech Laboratories, Inc., 3365), respectively. Recombinant proteins were indicated within BL21 (DE3) competent cells (Vazyme Biotech, C504-03), followed by purification for the GST affinity isolation analysis using the GST Protein Interaction Pull-Down Kit (Thermo, 21516) according to the instructions of the manufacturer. The bait proteins and prey proteins were subject to incubation with GST glutathione agarose resin. Then, the bound proteins were eluted through glutathione and explored with immunoblotting through the indicated antibodies.

### Confocal immunofluorescence assay

Cells were seeded on coverslips within six-well plates and transfected with indicated plasmids. The following day, the cells were fixed with 4% paraformaldehyde (Sigma-Aldrich, P6148) for a quarter and subsequently rinsed three times with PBS and permeabilized with 0.1% Triton X-100 (Sigma-Aldrich, T9284) for 10 min at room temperature. When the cells were rinsed three times with PBS, they were blocked with the concentration of 5% bovine serum albumin (Cell Signaling Technology, 9998) for 60 min at 37°C, followed by incubation with primary antibody for 60 min at 37°C. The cells were rinsed three times and subsequently incubated with a fluorescently labeled secondary antibody for 60 min at 37°C in the dark. When the cells were rinsed three times with PBS, they were stained with 4′,6-diamidino-2-phenylindole (Beyotime Biotechnology, C1002) at room temperature for 5 min. A confocal immunofluorescence microscope (Carl Zeiss, Oberkochen, Germany) was used for the observation of the cells.

### Statistical analysis

The obtained results are indicated as three independent experiments. The obtained values are shown to be means ± standard deviations and were explored using the two-tailed Student’s *t*-test with the GraphPad Prism 5 software (GraphPad Software, USA). *P*-values of <0.05 were considered to be of statistical significance.

## Data Availability

All data are contained within the article.
